# Orkney Springs, Virginia

**Published:** 1879-07

**Authors:** 


*Orkney Springs, Virginia.*—This old and popular watering place opens June 15th. To the broken down, or those with worn down energies, we know no better resort. Every dentist should have six weeks or two months' recreation, and the mineral springs of Virginia afford facilities for recuperation not excelled anywhere else. According to a late writer who makes his deductions from the last United States Census, the region of country marked by the mineral springs of Virginia enjoys greater immunity from pulmonary diseases than any other region of the Union. H.

